# Profiling tyrosine kinase inhibitors as AD therapeutics in a mouse model of AD

**DOI:** 10.1186/s13041-023-01051-9

**Published:** 2023-08-14

**Authors:** Hyun-ju Lee, Jeong-Woo Hwang, Jin-Hee Park, Yoo Joo Jeong, Ji-Yeong Jang, Hyang-Sook Hoe

**Affiliations:** 1 Department of Neural Development and Disease, Korea Brain Research Institute (KBRI), 61, Cheomdan-ro, Daegu, 41068 Republic of Korea; 2 Department of Brain and Cognitive Sciences, Daegu Gyeongbuk Institute of Science and Technology, Daegu, 42988 Republic of Korea

**Keywords:** Alzheimer’s disease, Tyrosine kinase inhibitors, Amyloid-β, Tau, Neuroinflammation, Ibrutinib, PD180970, Cabozantinib

## Abstract

Alzheimer’s disease (AD) is a neurodegenerative disease characterized by Aβ deposition, tauopathy, neuroinflammation, and impaired cognition. The recent identification of associations between protein kinases and AD pathology has spurred interest in tyrosine kinase inhibitors (TKIs) as potential strategic therapeutic agents for AD. In the present study, we investigated whether the TKIs ibrutinib, PD180970, and cabozantinib, which have different on-targets, selectively regulate AD pathology in 3.5- to 4-month-old 5xFAD mice (a model of the early phase of AD). Ibrutinib (10 mg/kg, i.p.) effectively reduced amyloid-β (Aβ) plaque number, tau hyperphosphorylation and neuroinflammation in 5xFAD mice. Surprisingly, PD180970 (10 mg/kg, i.p.) did not alter Aβ plaque number or neuroinflammatory responses and exacerbated tau hyperphosphorylation in 5xFAD mice. Cabozantinib (10 mg/kg, i.p.) had no effect on amyloidopathy but partially relieved tau hyperphosphorylation and astrogliosis. Taken together, our results suggest that not all TKIs have therapeutic effects on AD pathology in a mouse model of AD. Consequently, optimization of drug dosage, injection periods and administration routes should be considered when repurposing TKIs as novel AD therapeutics.

**Supplementary Information:**

The online version contains supplementary material available at 10.1186/s13041-023-01051-9.

Alzheimer’s disease (AD) is a neurodegenerative disease in which exacerbated deposition of senile plaques and neurofibrillary tangles and excessive neuroinflammation are followed by progressive memory deficits [1]. Importantly, several recent studies have implicated protein kinases in the exacerbation of AD pathoprogression, including amyloidogenesis, tauopathy, and neuronal death [2, 3]. These findings suggest that tyrosine kinase inhibitors (TKIs) could be leveraged as therapeutic agents for AD. We previously demonstrated that ibrutinib, a Bruton’s tyrosine kinase (BTK) inhibitor that is approved for treating chronic lymphocytic leukemia, has therapeutic effects on AD pathologies, including amyloid-β (Aβ) deposition, tauopathy, neuroinflammation, and cognitive function, in mouse models of AD [4]. We have also reported effects of other TKIs, including nilotinib, dasatinib, varlitinib, and vatalanib, on Aβ- and LPS-mediated AD pathologies [5–8]. However, whether all TKIs have therapeutic efficacy against AD pathoprogression is unknown.

In this study, we assessed the effects of PD180970, a TKI targeting breakpoint cluster region-Abelson murine leukemia (Bcr-Abl), and cabozantinib, a TKI targeting vascular endothelial growth factor receptor 2 (VEGFR2), on AD pathologies and compared their therapeutic efficacies with that of ibrutinib in 5xFAD mice, a mouse model of AD. PD180970 and cabozantinib were chosen because several studies have implicated VEGFR2 and c-Abl in the pathology of AD. For example, the active form of c-Abl, which contributes to tauopathy, is increased in the hippocampus in AD patients compared to healthy controls postmortem [9]. In addition, VEGF and VEGFR2 mRNA levels in the entorhinal cortex are elevated in an aged AD mouse model [10]. Moreover, we previously reported that inhibiting Bcr-Abl and VEGF significantly attenuates LPS- or Aβ-mediated neuroinflammation, Aβ/tau pathology, or cognitive impairment in wild-type mice and an AD mouse model [5–7]. Importantly, there is evidence that both cabozantinib and PD180970 can penetrate the brain. Cabozantinib crosses the blood–brain barrier (BBB) and exhibits therapeutic efficacy against brain metastasis in patients with renal cell carcinoma [11–13], and PD180970 attenuates MPTP-induced cytotoxicity by inducing autophagy in the brain in a Parkinsonian mouse model [14].

For TKI treatment, 3.5- to 4-month-old male 5xFAD mice (a model of the early phase of AD) were injected daily with vehicle (10% DMSO + 40% PEG + 5% Tween 80 + 45% saline, i.p.), ibrutinib (10 mg/kg, i.p.), PD180970 (10 mg/kg, i.p.), or cabozantinib (10 mg/kg, i.p.) for 14 consecutive days. Then, immunofluorescence staining of brain sections was performed to analyze the therapeutic effects of the TKIs on amyloidopathy, tau hyperphosphorylation, and gliosis.

Compared to vehicle treatment, ibrutinib significantly reduced 4G8-labeled Aβ plaque deposition in the cortex and subiculum in 5xFAD mice (Fig. 1A–E), whereas PD180970 and cabozantinib had no significant effects on Aβ plaque accumulation in the cortex and subiculum (Fig. 1B–E). Next, we investigated whether the TKIs influence Aβ pathology by modulating the Aβ-degrading enzyme neprilysin (NEP) in 5xFAD mice. Ibrutinib significantly increased NEP expression in the cortex and subiculum compared to vehicle-treated group, and in the subiculum compared to PD1810970 (Fig. 1F–I). However, PD180970 and cabozantinib did not affect NEP levels in the cortex and subiculum (Fig. 1F–I). These data indicated that ibrutinib ameliorates amyloid pathology by promoting the expression of the Aβ-degrading enzyme NEP in this early-phase AD mouse model, whereas PD180970 and cabozantinib have no effect.

**Fig. 1:**
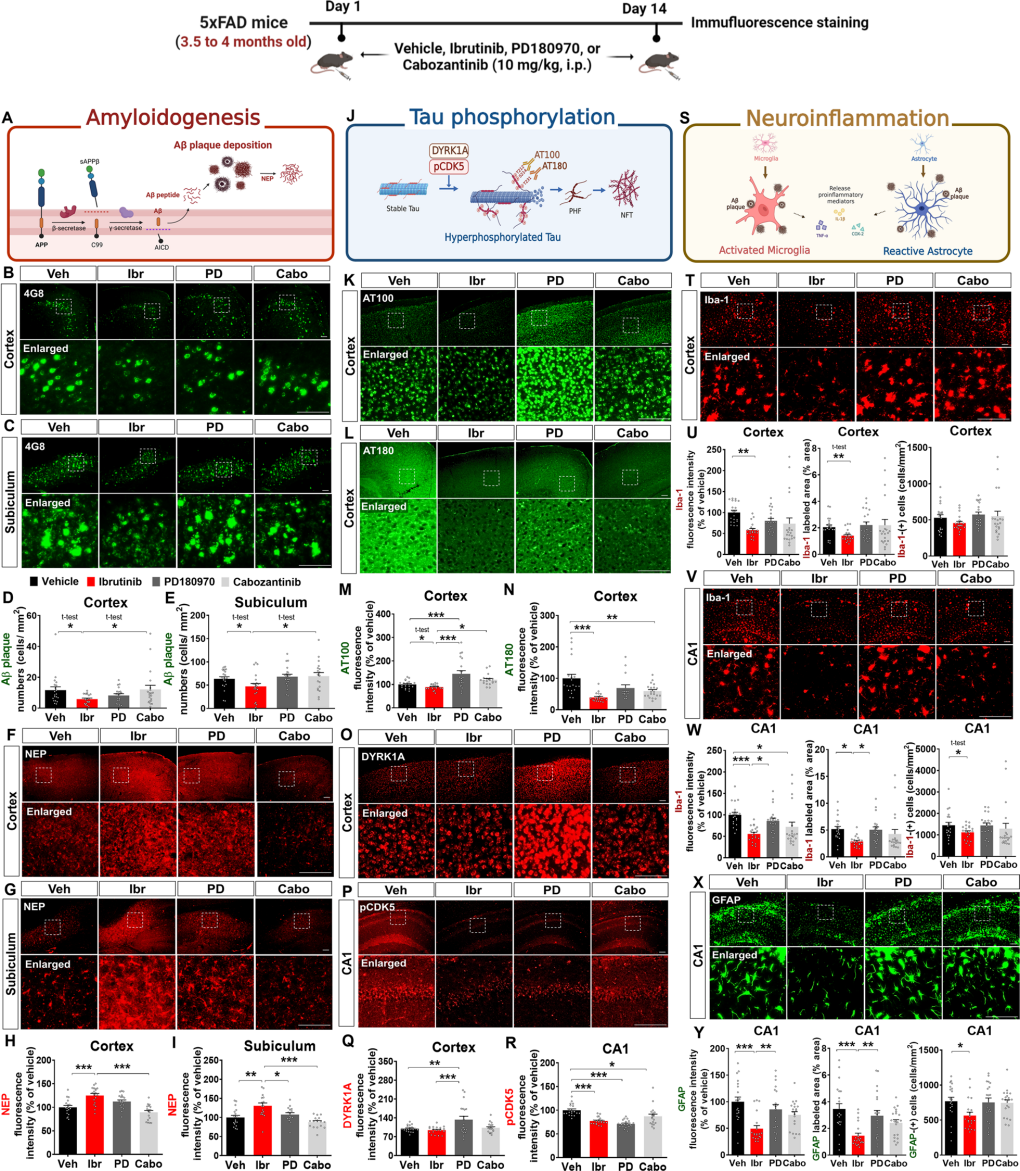
Comparison of the therapeutic effects of TKIs on AD pathology in 5xFAD mice. **A** Schematic illustration of amyloid pathology.** B**, **C** Immunofluorescence staining of 4G8 in brain slices from 5xFAD mice injected daily with vehicle (10% DMSO + 40% PEG300 + 5% Tween80 + 45% saline), ibrutinib (10 mg/kg, i.p.), PD180970 (10 mg/kg, i.p.), or cabozantinib (10 mg/kg, i.p.) for 14 consecutive days. **D**, **E** Quantification of data from B-C (n = 18–21 brain slices from 3–5 mice/group). **F**, **G** Immunofluorescence staining of NEP in brain slices from 5xFAD mice injected daily with vehicle, ibrutinib, PD180970, or cabozantinib for 14 consecutive days. **H**, **I** Quantification of data from F-G (n = 16–20 brain slices from 3–5 mice/group).** J** Schematic illustration of tau hyperphosphorylation. **K**–**L** Immunofluorescence staining of AT100 (K) or AT180 (L) in brain slices from 5xFAD mice injected daily with vehicle, ibrutinib, PD180970, or cabozantinib for 14 consecutive days. **M** Quantification of data from K (n = 19–20 brain slices from 4–5 mice/group). **N** Quantification of data from L (n = 15–20 brain slices from 4–5 mice/group). **O**, **P** Immunofluorescence staining of DYRK1A (O) or p-CDK5 (P) in brain slices from 5xFAD mice injected daily with vehicle, ibrutinib, PD180970, or cabozantinib for 14 consecutive days. **Q** Quantification of data from O (n = 19–20 brain slices from 4–5 mice/group). **R** Quantification of data from P (n = 19–20 brain slices from 4–5 mice/group). **S** Schematic illustration of neuroinflammation. **T**, **V** Immunofluorescence staining of Iba-1 in brain slices from 5xFAD mice injected daily with vehicle, ibrutinib, PD180970, or cabozantinib for 14 consecutive days. **U**, **W** Quantification of data from T and V, respectively (n = 20 brain slices from 4–5 mice/group). **X** Immunofluorescence staining of GFAP in brain slices from 5xFAD mice injected daily with vehicle, ibrutinib, PD180970, or cabozantinib for 14 consecutive days. **Y** Quantification of data from X (n = 20 brain slices from 4–5 mice/group). *p < 0.05, **p < 0.01, and ***p < 0.001. Scale bar = 100 μm

The analysis of tau hyperphosphorylation showed that ibrutinib significantly reduced phospho-tau^Thr212/Ser214^ (detected by AT100) levels in the cortex in 5xFAD mice compared to vehicle, PD180970 and cabozantinib (Fig. 1K, M). Unexpectedly, PD180970 significantly increased phospho-tau^Thr212/Ser214^ levels in the cortex in 5xFAD mice compared to vehicle, whereas cabozantinib had no effect (Fig. 1K, M). Ibrutinib also significantly decreased phospho-tau^Thr231^ (detected by AT180) levels in the cortex (Fig. 1L, N) but not in the hippocampal CA1 region (Additional file 1: Fig. S1). In addition, PD180970 did not alter phospho-tau^Thr231^ levels in the brain in 5xFAD mice compared with vehicle, whereas cabozantinib significantly diminshed phospho-tau^Thr231^ levels in the cortex in 5xFAD mice (Fig. 1L, N and Additional file 1: Fig. S1). These data indicate that ibrutinib suppresses tau hyperphosphorylation, PD180970 exacerbates tauopathy, and cabozantinib partially ameliorates tau hyperphosphorylation in the early-phase AD mouse model.

Since tau kinases are responsible for tau hyperphosphorylation, we further investigated the effects of the TKIs on tau kinase expression and activity. Ibrutinib did not modulate the levels of dual specificity tyrosine phosphorylation-regulated kinase 1A (DYRK1A) in 5xFAD mice compared with vehicle (Fig. 1O, Q and Additional file 1: Fig. S2). Consistent with the increase in tau hyperphosphorylation (Fig. 1K, M), PD180970 significantly upregulated DYRK1A levels in the cortex (Fig. 1O, Q) and hippocampal CA1 region (Additional file 1: Fig.S2) in 5xFAD mice compared with vehicle and ibrutinib treatment. Cabozantinib did not affect DYRK1A levels in the cortex in 5xFAD mice compared with vehicle (Fig. 1O, Q) but significantly increased DYRK1A levels in the hippocampal CA1 region compared with ibrutinib treatment (Additional file 1: Fig. S2). Moreover, ibrutinib, PD180970, and cabozantinib significantly suppressed the levels of another tau kinase, phospho-cyclin dependent kinase 5 (p-CDK5), in the hippocampal CA1 region in 5xFAD mice (Fig. 1P, R). How do these TKIs downregulate the phosphorylation of the tau kinase CDK5 in 5xFAD mice despite either upregulating or not affecting the tau kinase DYRK1A? Ibrutinib, PD180970, and cabozantinib have different on-targets (BTK, Bcr-Abl, and VEGFR2, respectively) and off-targets, but all were developed to treat cancer. Most anticancer drugs suppress the unregulated cell cycle, and CDK5 is involved in the cell cycle checkpoint and oncogenesis [15, 16]. The specific mechanisms by which TKIs differentially modulate tau kinases in mouse models of AD (5xFAD and Tau Tg PS19) will be examined in future work. Overall, our data from this early-phase AD mouse model suggest that ibrutinib ameliorates tau hyperphosphorylation by downregulating tau kinase CDK5 phosphorylation, PD180970 enhances tau hyperphosphorylation by upregulating the tau kinase DYRK1A, and cabozantinib partially attenuates tauopathy by suppressing CDK5 phosphorylation.

Amyloidopathy and tauopathy are closely associated with neuroinflammation, which is predominantly regulated by microglia and astrocytes [17–19]. Therefore, we determined whether the TKIs modulate microglia-associated neuroinflammation in 5xFAD mice. Ibrutinib diminished Iba-1 fluorescence intensity and microglial hypertrophy (% of Iba-1-labeled area) in the cortex and hippocampal CA1 region but decreased the number of Iba-1-positive microglia only in the hippocampal CA1 region  compared with vehicle-treated group (Fig. 1T–W). Moreover, PD180970 significantly increased Iba-1 fluorescence intensity and % of Iba-1-labeled area compared with ibrutinib in CA1 of 5xFAD mice (Fig. 1T–W). In addition, cabozantinib only downregulated Iba-1 fluorescence intensity in the hippocampal CA1 region (Fig. 1V–W). These data indicate that ibrutinib attenuates microglial activation, PD180970 has no effect, and cabozantinib partially reduces microgliosis in 5xFAD mice.

Finally, we examined the effects of the TKIs on astrocyte-associated neuroinflammation in 5xFAD mice. Ibrutinib significantly diminished GFAP fluorescence intensity, astrocytic hypertrophy (% of GFAP-labeled area), and the number of GFAP-positive astrocytes compared to vehicle-treated group in the hippocampal CA1 region (Fig. 1X, Y). However, PD180970 and cabozantinib did not modulate astrogliosis in 5xFAD mice (Fig. 1X, Y). Taken together, the data from the early-phase AD mouse model suggest that ibrutinib ameliorates astrocyte-mediated neuroinflammation, whereas PD180970 and cabozantinib have no effects on astrogliosis.

In conclusion, ibrutinib effectively attenuated amyloidogenesis, tau hyperphosphorylation, and microglia/astrocyte-associated neuroinflammation in the early-phase AD mouse model, consistent with our previous findings [4]. A limitation of the present study is that we did not examine how these mechanisms influence/interact with each other to downregulate AD pathologies. Their potential synergistic effects could be sequential or reciprocal. Future studies will unveil the specific relationships between these mechanisms on AD pathology. In contrast to the therapeutic efficacy of ibrutinib, PD180970 did not alter amyloidogenesis or neuroinflammation but exacerbated tau hyperphosphorylation in 5xFAD mice. Cabozantinib had no effect on amyloidopathy but partially relieved tauopathy and astrogliosis. Most strikingly, PD180970 had no effect on AD pathology, even though the on-target of PD180970 is identical to those of the Bcr-Abl kinase inhibitors nilotinib and dasatinib, which alleviate the LPS-mediated neuroinflammatory response and AD pathology [5, 6]. Our findings raise the question of why these TKIs have differential therapeutic efficacies against AD pathologies. It is possible that off-target effects obscure on-target therapeutic efficacy against AD. Another possibility is that the optimal dose, injection period, or injection route for AD treatment varies among TKIs. Daily intraperitoneal injection of the TKIs at a dose of 10 mg/kg for 14 days may not have been sufficient for some of the TKIs to ameliorate early AD symptoms in 5xFAD mice. The treatment regimens (dosage and administration route/periods) for PD180970 and cabozantinib in AD mouse models will be optimized in future work. Taken together, our results suggest that TKIs, which have been proposed as potential strategic therapeutic agents for ameliorating AD pathologies, can positively or negatively modulate AD pathoprogression in this mouse model of the early phase of AD. Therefore, when repurposing TKIs as novel AD therapeutics, on- and off-target effects, the mode of action under pathological conditions (AD), and optimization of dose, periods, and routes should be considered.

### Supplementary Information

**Additional file 1: Fig S1.** Ibrutinib, PD180970, and cabozantinib do not alter tau phosphorylation at residue Thr231 in the hippocampal CA1 region in 3.5- to 4-month-old 5xFAD mice. **A** Immunofluorescence staining of AT180 in brain slices from 5xFAD mice injected daily with vehicle (10% DMSO+40% PEG300+5% Tween80+45% saline), ibrutinib (10 mg/kg, i.p.), PD180970 (10 mg/kg, i.p.), or cabozantinib (10 mg/kg, i.p.) for 14 consecutive days. **B** Quantification of data from A (n=15–19 brain slices from 3–5 mice/group). Scale bar=100 μm. **Fig S2.** PD180970 upregulates the tau kinase DYRK1A in the hippocampal CA1 region in 3.5- to 4-month-old 5xFAD mice. **A** Immunofluorescence staining of DYRK1A in brain slices from 5xFAD mice injected daily with vehicle (10% DMSO+40% PEG300 + 5% Tween80 + 45% saline), ibrutinib (10 mg/kg, i.p.), PD180970 (10 mg/kg, i.p.), or cabozantinib (10 mg/kg, i.p.) for 14 consecutive days. **B** Quantification of data from A (n=19-20 brain slices from 4–5 mice/group). *p<0.05, **p<0.01, Scale bar=100 μm.

## Data Availability

All data generated and/or analyzed during this study are included in this published article and its supplementary materials. Materials and methods are presented in the supplementary materials.

## Abbreviations

AD: Alzheimer’s disease
TKI: Tyrosine kinase inhibitor
BTK: Bruton’s tyrosine kinase
JAK: Janus kinase
Bcr-Abl: Breakpoint cluster region-Abelson murine leukemia
VEGFR2: Vascular endothelial growth factor receptor 2
Aβ: Amyloid-β
CDK-5: Cyclin dependent kinase-5
DYRK1A: Dual specificity tyrosine-phosphorylation-regulated kinase 1A
