# Supplementary material for: Profiling tyrosine kinase inhibitors as AD therapeutics in a mouse model of AD
Source: Mol Brain. 2023 Aug 14;16:63. doi: 10.1186/s13041-023-01051-9 (PMC10426186; doi:10.1186/s13041-023-01051-9)
Supplement: Supplementary file 1 — Additional file 1: Fig S1. Ibrutinib, PD180970, and cabozantinib do not alter tau phosphorylation at residue Thr231 in the hippocampal CA1 region in 3.5- to 4-month-old 5xFAD mice. A Immunofluorescence staining of AT180 in brain slices from 5xFAD mice injected daily with vehicle (10% DMSO+40% PEG300+5% Tween80+45% saline), ibrutinib (10 mg/kg, i.p.), PD180970 (10 mg/kg, i.p.), or cabozantinib (10 mg/kg, i.p.) for 14 consecutive days. B Quantification of data from A (n=15–19 brain slices from 3–5 mice/group). Scale bar=100 μm. Fig S2. PD180970 upregulates the tau kinase DYRK1A in the hippocampal CA1 region in 3.5- to 4-month-old 5xFAD mice. A Immunofluorescence staining of DYRK1A in brain slices from 5xFAD mice injected daily with vehicle (10% DMSO+40% PEG300 + 5% Tween80 + 45% saline), ibrutinib (10 mg/kg, i.p.), PD180970 (10 mg/kg, i.p.), or cabozantinib (10 mg/kg, i.p.) for 14 consecutive days. B Quantification of data from A (n=19-20 brain slices from 4–5 mice/group). *p<0.05, **p<0.01, Scale bar=100 μm. [file 13041_2023_1051_MOESM1_ESM.docx]

**Additional file 1**

**Profiling tyrosine kinase inhibitors as AD therapeutics in a mouse model of AD**

**Hyun-ju Lee^1,*^, Jeong-Woo Hwang^1^, Jin-Hee Park^1,2^, Yoo Joo Jeong^1,2^, Ji-Yeong Jang^1^, Hyang-Sook Hoe^1,2,*^**

^1^ Department of Neural Development and Disease, Korea Brain Research Institute (KBRI), 61, Cheomdan-ro, Daegu, 41068 Republic of Korea; ^2^ Department of Brain and Cognitive Sciences, Daegu Gyeongbuk Institute of Science & Technology, Daegu 42988, Republic of Korea

* Corresponding authorrs:

**Hyang-Sook Hoe**, PhD: Department of Neural Development and Disease, Korea Brain Research Institute (KBRI), 61, Cheomdan-ro, Daegu, Republic of Korea, Tel: 82-53-980-8310, Fax: 85-53-980-8309, E-mail: [sookhoe72@kbri.re.kr](mailto:sookhoe72@kbri.re.kr)

**Hyun-ju Lee**, PhD: Department of Neural Development and Disease, Korea Brain Research Institute (KBRI), 61, Cheomdan-ro, Daegu, Republic of Korea, Tel: 82-53-980-8313, Fax: 85-53-980-8309, E-mail: [hjlee@kbri.re.kr](mailto:hjlee@kbri.re.kr)

**
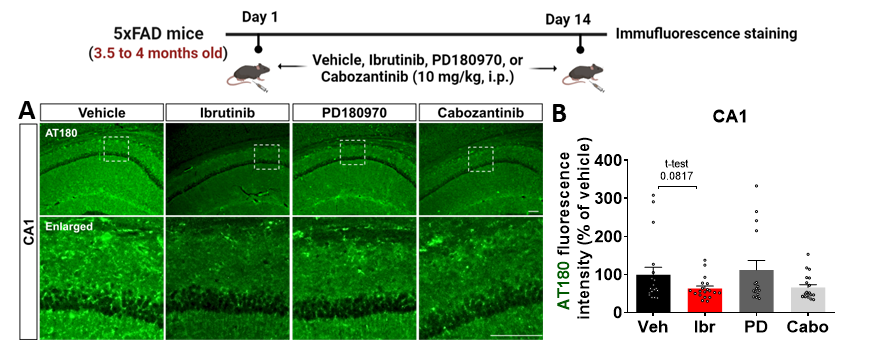
****Fig S1. Ibrutinib, PD180970, and cabozantinib do not alter tau phosphorylation at residue Thr231 in the hippocampal CA1 region in 3.5- to 4-month-old 5xFAD mice. (A)** Immunofluorescence staining of AT180 in brain slices from 5xFAD mice injected daily with vehicle (10% DMSO + 40% PEG300 + 5% Tween80 + 45% saline), ibrutinib (10 mg/kg, i.p.), PD180970 (10 mg/kg, i.p.), or cabozantinib (10 mg/kg, i.p.) for 14 consecutive days. (**B)** Quantification of data from A (n = 15-19 brain slices from 3-5 mice/group). Scale bar = 100 μm.

**
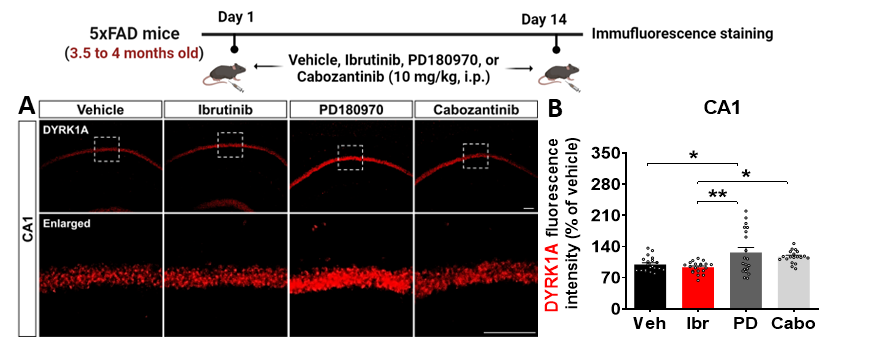
Fig S2. PD180970 upregulates the tau kinase DYRK1A in the hippocampal CA1 region in 3.5- to 4-month-old 5xFAD mice. (A)** Immunofluorescence staining of DYRK1A in brain slices from 5xFAD mice injected daily with vehicle (10% DMSO + 40% PEG300 + 5% Tween80 + 45% saline), ibrutinib (10 mg/kg, i.p.), PD180970 (10 mg/kg, i.p.), or cabozantinib (10 mg/kg, i.p.) for 14 consecutive days. (**B)** Quantification of data from A (n = 19-20 brain slices from 4-5 mice/group). **p* < 0.05, ***p* < 0.01, Scale bar = 100 μm.

**Materials and methods**

**Mice**

The effects of tyrosine kinase inhibitors (TKIs) on AD pathology were evaluated in Aβ-overexpressing 5xFAD mice (3.5 to 4 months old, B6Cg-Tg APPSwFlLon, PSEN1*M146L*L286V6799Vas/Mmjax; stock # 34848-JAX, Jackson Laboratory, Bar Harbor, ME, USA). The mice were maintained in a pathogen-free facility with a 12-hour light/dark cycle (lights on at 8 a.m.) in cages housing 3–5 mice each and had access to food and water ad libitum. All in vivo experiments were approved by the institutional biosafety committee (IBC) and were performed in accordance with approved animal protocols and guidelines established by the institutional animal care and use committee of Korea Brain Research Institute (KBRI, approval numbers: IACUC-19–00049 and IACUC-22–00044).

**Drug preparation and administration**

Ibrutinib (S2680, Selleck Chemicals, Houston, TX, USA), PD180970 (1137, Axon Medchem, Groningen, Groningen, the Netherlands), and cabozantinib (HY-13016, MedChemExpress, Monmouth Junction, NJ, USA) were prepared in vehicle (10% DMSO + 40% PEG + 5% Tween 80 + 45% saline). To investigate the therapeutic efficacy of PD180970 and cabozantinib compared with that of ibrutinib, the drug dosage, injection route, and injection period were identical to those used for ibrutinib administration in our previous study [1]. Therefore, male 5xFAD mice (3.5–4 months old) were intraperitoneally (i.p.) injected with ibrutinib (10 mg/kg), PD180970 (10 mg/kg), cabozantinib (10 mg/kg), or vehicle daily for 14 consecutive days. According to the information provided by the manufacturer (https://www.medchemexpress.com), the solubilities of ibrutinib and cabozantinib in the vehicle formulation are ≥ 2.5 mg/ml and ≥ 2.08 mg/ml, respectively. Detailed solubility information is provided in Supplementary Table 1. We found that all TKIs used in the present study were clearly dissolved in the vehicle formulation at the dosage of 10 mg/kg.

**Immunofluorescence staining**

To evaluate the effects of the TKIs on AD pathology, 5xFAD mice that had been treated with TKIs or vehicle were anesthetized with 2,2,2-tribromoethanol (Sigma Aldrich, St. Louis, MO, USA, 2.5% v/v, 150 mg/kg, i.p.) and transcardially perfused sequentially with PBS and ice-cold 4% PFA (Chembio, Seoul, Republic of Korea). The brains were removed and post-fixed sequentially in 4% paraformaldehyde overnight and PBS containing 30% sucrose (w/v) for 3 days at 4 °C. Coronal brain slices with a thickness of 35 µm were obtained by using a cryostat (Leica CM1850, Wetzlar, Germany). Free floating sections were permeabilized with PBS containing 0.2% Triton X-100 (PBST) and 10% normal goat serum at room temperature for 1 h and then incubated with one of the following primary antibodies: anti-4G8 (1:500; 800704, BioLegend, San Diego, CA, USA), anti-NEP (1:400, AB5458, Millipore, Burlington, MS, USA), anti-AT100 (1:100; MN1060, Invitrogen, Carlsbad, CA, USA), anti-AT180 (1:200; MN1040, Invitrogen, Carlsbad, CA, USA), anti-DYRK1A (1:200; AB180910, Abcam, Cambridge, UK), anti-p-CDK5^Tyr15^ (1:200; LS‑C354604, LSbio, Seattle, WA, USA), anti-Iba-1 (1:500; 019-19741, Wako, Chouku, Osaka, Japan), or anti-GFAP (1:500; 13-0300, Invitrogen, Carlsbad, CA, USA). After incubation with the primary antibody, the sections were washed with PBST three times and incubated with an appropriate secondary antibody at room temperature for 2 h: anti-rabbit, anti-mouse, or anti-rat conjugated with Alexa Fluor 488 or 594. After washing three times with PBS, the sections were mounted in antifade mounting medium containing DAPI (Vector Laboratories, Burlingame, CA, USA). Finally, the sections were imaged by fluorescence microscopy (DMi8, Leica Microsystems, Wetzlar, Germany), and the images were analyzed by ImageJ (version 1.53a, National Institutes of Health, Bethesda, MD, USA).

**Quantification of immunofluorescence-stained images**

To measure the number of 4G8-positive Aβ plaques in the cortex and subiculum in 5xFAD mice, designated brain regions were selected as regions of interest (ROIs) in DAPI-stained images. Then, the extracellular Aβ plaques accumulated in the ROIs were manually counted, and the number of Aβ plaques per mm^2^ was calculated. To quantify the fluorescence intensities of NEP, AT180, AT100, pCDK5, DYRK1A, Iba-1, and GFAP in the cortex and/or hippocampal CA1 region, specific brain regions were designated as ROIs in DAPI-stained images, and the fluorescence intensity in the designated ROIs was determined semi-automatically using ImageJ software (version 1.53a, U. S. National Institutes of Health, Bethesda, ME, USA). To assess the fraction of Iba-1- or GFAP-labeled area in the cortex and/or hippocampal CA1 region, specific brain regions were designated as ROIs in DAPI-stained images, the immunoreactive area was classified by thresholding, and the immunopositive percentage of the total area was calculated. In addition, the number of Iba-1- or GFAP-positive glial cells in the ROIs was semi-automatically counted using ImageJ software.

**Statistical analysis**

For multiple comparisons, one-way analysis of variance (ANOVA) followed by Tukey’s post hoc analysis was used. For comparisons between two groups, the two-tailed unpaired *t*-test was used. GraphPad Prism 8 software (GraphPad Software, San Diego, CA, USA) was used to generate graphs and to perform statistical analysis. Individual data points and means ± SEMs are presented. Asterisks indicate significance: * p <0.05, ** p < 0.01, and *** p < 0.001.

**Supplementary Table 1.** Solubility of ibrutinib, PD180970, and cabozantinib.

| **Drugs** | **Solvent** | **Solubility** | **References** |
| --- | --- | --- | --- |
| **Ibrutinib** | 5% DMSO + 40% PEG 300 + 5% Tween 80 + 45% saline | ≥ 2.5 mg/ml | https://www.medchemexpress.com/PCI-32765.html |
|  | 10% DMSO + 90% corn oil | ≥ 2.5 mg/ml |  |
| **PD180970** | 10% DMSO + 90% corn oil | ≥ 1.25 mg/ml | https://www.medchemexpress.com/pd180970.html |
| **Cabozantinib** | 5% DMSO + 40% PEG 300 + 5% Tween 80 + 45% saline | ≥ 2.08 mg/ml | https://www.medchemexpress.com/Cabozantinib.html |
|  | 10% DMSO + 90% corn oil | ≥ 2.08 mg/ml |  |

**Supplementary Table 2.** Statistical analysis results of the in vivo experiments.

| **Figure 1D. 4G8-positive Aβ plaque numbers - Cortex** |
| --- |
| \| \| Table Analyzed \| Abeta plaque number cortex \| \| --- \| --- \| \|  \|  \| \| Column B \| Ibr \| \| vs. \| vs. \| \| Column A \| Veh \| \|  \|  \| \| Unpaired t test \|  \| \| P value \| 0.0171 \| \| P value summary \| * \| \| Significantly different (P < 0.05)? \| Yes \| \| One- or two-tailed P value? \| Two-tailed \| \| t, df \| t=2.493, df=38 \|  \| Table Analyzed \| Abeta plaque number cortex \| \| --- \| --- \| \|  \|  \| \| Column D \| Cabo \| \| vs. \| vs. \| \| Column B \| Ibr \| \|  \|  \| \| Unpaired t test \|  \| \| P value \| 0.0292 \| \| P value summary \| * \| \| Significantly different (P < 0.05)? \| Yes \| \| One- or two-tailed P value? \| Two-tailed \| \| t, df \| t=2.266, df=38 \| \|  \| \| --- \| --- \| --- \| --- \| --- \| --- \| --- \| --- \| --- \| --- \| --- \| --- \| --- \| --- \| --- \| --- \| --- \| --- \| --- \| --- \| --- \| --- \| --- \| --- \| --- \| --- \| --- \| --- \| --- \| --- \| --- \| --- \| --- \| --- \| --- \| --- \| --- \| --- \| --- \| --- \| --- \| --- \| --- \| --- \| --- \| --- \| --- \| --- \| --- \| --- \| |
| **Figure 1E. 4G8-positive Aβ plaque numbers - Subiculum** |
| \| \| Table Analyzed \| Abeta plaque number subi \| \| --- \| --- \| \|  \|  \| \| Column B \| Ibr \| \| vs. \| vs. \| \| Column A \| Veh \| \|  \|  \| \| Unpaired t test \|  \| \| P value \| 0.0421 \| \| P value summary \| * \| \| Significantly different (P < 0.05)? \| Yes \| \| One- or two-tailed P value? \| Two-tailed \| \| t, df \| t=2.102, df=39 \|  \| Table Analyzed \| Abeta plaque number subi \| \| --- \| --- \| \|  \|  \| \| Column D \| Cabo \| \| vs. \| vs. \| \| Column B \| Ibr \| \|  \|  \| \| Unpaired t test \|  \| \| P value \| 0.0309 \| \| P value summary \| * \| \| Significantly different (P < 0.05)? \| Yes \| \| One- or two-tailed P value? \| Two-tailed \| \| t, df \| t=2.246, df=36 \| \|  \| \| --- \| --- \| --- \| --- \| --- \| --- \| --- \| --- \| --- \| --- \| --- \| --- \| --- \| --- \| --- \| --- \| --- \| --- \| --- \| --- \| --- \| --- \| --- \| --- \| --- \| --- \| --- \| --- \| --- \| --- \| --- \| --- \| --- \| --- \| --- \| --- \| --- \| --- \| --- \| --- \| --- \| --- \| --- \| --- \| --- \| --- \| --- \| --- \| --- \| --- \| |
| **Figure 1H. NEP fluorescence intensity - Cortex** |
| \| \| Table Analyzed \| \| \| NEP-cortex \| \| \| --- \| --- \| --- \| --- \| --- \| \| Data sets analyzed \| \| \| A-D \| \| \|  \| \| \|  \| \| \| ANOVA summary \| \| \|  \| \| \| F \| \| \| 16.07 \| \| \| P value \| \| \| <0.0001 \| \| \| P value summary \| \| \| **** \| \| \| Significant diff. among means (P < 0.05)? \| \| \| Yes \| \| \| R square \| \| \| 0.4044 \| \| \| Number of families \| 1 \|  \| \|  \| \|  \|  \|  \| \| Number of comparisons per family \| 6 \|  \| \|  \| \|  \|  \|  \| \| Alpha \| 0.05 \|  \| \|  \| \|  \|  \|  \| \|  \|  \|  \| \|  \| \|  \|  \|  \| \| Tukey's multiple comparisons test \| Mean Diff. \| 95.00% CI of diff. \| \| Significant? \| \| Summary \| Adjusted P Value \|  \| \| Veh vs. Ibr \| -25.07 \| -39.38 to -10.75 \| \| Yes \| \| *** \| 0.0001 \| A-B \| \| Veh vs. PD \| -12.33 \| -26.47 to 1.812 \| \| No \| \| ns \| 0.1092 \| A-C \| \| Veh vs. Cabo \| 10.54 \| -3.963 to 25.05 \| \| No \| \| ns \| 0.2323 \| A-D \| \| Ibr vs. PD \| 12.74 \| -1.200 to 26.68 \| \| No \| \| ns \| 0.0854 \| B-C \| \| Ibr vs. Cabo \| 35.61 \| 21.30 to 49.93 \| \| Yes \| \| **** \| <0.0001 \| B-D \| \| PD vs. Cabo \| 22.87 \| 8.731 to 37.01 \| \| Yes \| \| *** \| 0.0004 \| C-D \| \|  \| \| --- \| --- \| --- \| --- \| --- \| --- \| --- \| --- \| --- \| --- \| --- \| --- \| --- \| --- \| --- \| --- \| --- \| --- \| --- \| --- \| --- \| --- \| --- \| --- \| --- \| --- \| --- \| --- \| --- \| --- \| --- \| --- \| --- \| --- \| --- \| --- \| --- \| --- \| --- \| --- \| --- \| --- \| --- \| --- \| --- \| --- \| --- \| --- \| --- \| --- \| --- \| --- \| --- \| --- \| --- \| --- \| --- \| --- \| --- \| --- \| --- \| --- \| --- \| --- \| --- \| --- \| --- \| --- \| --- \| --- \| --- \| --- \| --- \| --- \| --- \| --- \| --- \| --- \| --- \| --- \| --- \| --- \| --- \| --- \| --- \| --- \| --- \| --- \| --- \| --- \| --- \| --- \| --- \| --- \| --- \| --- \| --- \| --- \| --- \| --- \| --- \| --- \| --- \| --- \| --- \| --- \| --- \| --- \| --- \| --- \| --- \| --- \| --- \| --- \| --- \| --- \| --- \| --- \| --- \| --- \| --- \| --- \| --- \| --- \| --- \| --- \| --- \| --- \| --- \| --- \| --- \| --- \| --- \| --- \| --- \| --- \| --- \| --- \| --- \| --- \| --- \| --- \| --- \| --- \| --- \| --- \| |
| **Figure 1I. NEP fluorescence intensity - Subiculum** |
| \| \| Table Analyzed \| \| \| NEP-subiculum \| \| \| --- \| --- \| --- \| --- \| --- \| \| Data sets analyzed \| \| \| A-D \| \| \|  \| \| \|  \| \| \| ANOVA summary \| \| \|  \| \| \| F \| \| \| 10.01 \| \| \| P value \| \| \| <0.0001 \| \| \| P value summary \| \| \| **** \| \| \| Significant diff. among means (P < 0.05)? \| \| \| Yes \| \| \| R square \| \| \| 0.3262 \| \| \| Number of families \| 1 \|  \| \|  \| \|  \|  \|  \| \| Number of comparisons per family \| 6 \|  \| \|  \| \|  \|  \|  \| \| Alpha \| 0.05 \|  \| \|  \| \|  \|  \|  \| \|  \|  \|  \| \|  \| \|  \|  \|  \| \| Tukey's multiple comparisons test \| Mean Diff. \| 95.00% CI of diff. \| \| Significant? \| \| Summary \| Adjusted P Value \|  \| \| Veh vs. Ibr \| -30.56 \| -51.30 to -9.832 \| \| Yes \| \| ** \| 0.0014 \| A-B \| \| Veh vs. PD \| -7.565 \| -28.30 to 13.17 \| \| No \| \| ns \| 0.7706 \| A-C \| \| Veh vs. Cabo \| 12.27 \| -8.456 to 33.01 \| \| No \| \| ns \| 0.4069 \| A-D \| \| Ibr vs. PD \| 23.00 \| 1.666 to 44.33 \| \| Yes \| \| * \| 0.0298 \| B-C \| \| Ibr vs. Cabo \| 42.84 \| 21.51 to 64.17 \| \| Yes \| \| **** \| <0.0001 \| B-D \| \| PD vs. Cabo \| 19.84 \| -1.492 to 41.17 \| \| No \| \| ns \| 0.0773 \| C-D \| \|  \| \| --- \| --- \| --- \| --- \| --- \| --- \| --- \| --- \| --- \| --- \| --- \| --- \| --- \| --- \| --- \| --- \| --- \| --- \| --- \| --- \| --- \| --- \| --- \| --- \| --- \| --- \| --- \| --- \| --- \| --- \| --- \| --- \| --- \| --- \| --- \| --- \| --- \| --- \| --- \| --- \| --- \| --- \| --- \| --- \| --- \| --- \| --- \| --- \| --- \| --- \| --- \| --- \| --- \| --- \| --- \| --- \| --- \| --- \| --- \| --- \| --- \| --- \| --- \| --- \| --- \| --- \| --- \| --- \| --- \| --- \| --- \| --- \| --- \| --- \| --- \| --- \| --- \| --- \| --- \| --- \| --- \| --- \| --- \| --- \| --- \| --- \| --- \| --- \| --- \| --- \| --- \| --- \| --- \| --- \| --- \| --- \| --- \| --- \| --- \| --- \| --- \| --- \| --- \| --- \| --- \| --- \| --- \| --- \| --- \| --- \| --- \| --- \| --- \| --- \| --- \| --- \| --- \| --- \| --- \| --- \| --- \| --- \| --- \| --- \| --- \| --- \| --- \| --- \| --- \| --- \| --- \| --- \| --- \| --- \| --- \| --- \| --- \| --- \| --- \| --- \| --- \| --- \| --- \| --- \| --- \| --- \| |
| **Figure 1M. AT100 fluorescence intensity - Cortex** |
| \| Table Analyzed \| AT100-cortex \| \| --- \| --- \| \|  \|  \| \| Column B \| Ibr \| \| vs. \| vs. \| \| Column A \| Veh \| \|  \|  \| \| Unpaired t test \|  \| \| P value \| 0.0184 \| \| P value summary \| * \| \| Significantly different (P < 0.05)? \| Yes \| \| One- or two-tailed P value? \| Two-tailed \| \| t, df \| t=2.464, df=38 \|  \| Table Analyzed \| \| \| AT100-cortex \| \| --- \| --- \| --- \| --- \| \| Data sets analyzed \| \| \| A-D \| \|  \| \| \|  \| \| ANOVA summary \| \| \|  \| \| F \| \| \| 10.16 \| \| P value \| \| \| <0.0001 \| \| P value summary \| \| \| **** \| \| Significant diff. among means (P < 0.05)? \| \| \| Yes \| \| R square \| \| \| 0.2891 \| \| Number of families \| 1 \|  \| \| \|  \|  \|  \|  \| \| Number of comparisons per family \| 6 \|  \| \| \|  \|  \|  \|  \| \| Alpha \| 0.05 \|  \| \| \|  \|  \|  \|  \| \|  \|  \|  \| \| \|  \|  \|  \|  \| \| Tukey's multiple comparisons test \| Mean Diff. \| 95.00% CI of diff. \| \| \| Significant? \| Summary \| Adjusted P Value \|  \| \| Veh vs. Ibr \| 11.23 \| -17.56 to 40.03 \| \| \| No \| ns \| 0.7353 \| A-B \| \| Veh vs. PD \| -45.08 \| -73.88 to -16.29 \| \| \| Yes \| *** \| 0.0006 \| A-C \| \| Veh vs. Cabo \| -19.95 \| -49.12 to 9.221 \| \| \| No \| ns \| 0.2829 \| A-D \| \| Ibr vs. PD \| -56.32 \| -85.11 to -27.52 \| \| \| Yes \| **** \| <0.0001 \| B-C \| \| Ibr vs. Cabo \| -31.18 \| -60.35 to -2.011 \| \| \| Yes \| * \| 0.0315 \| B-D \| \| PD vs. Cabo \| 25.13 \| -4.036 to 54.30 \| \| \| No \| ns \| 0.1158 \| C-D \| |
| **Figure 1N. AT180 fluorescence intensity - Cortex** |
| \| Table Analyzed \| \| AT180-cortex \| \| \| --- \| --- \| --- \| --- \| \| Data sets analyzed \| \| A-D \| \| \|  \| \|  \| \| \| ANOVA summary \| \|  \| \| \| F \| \| 9.350 \| \| \| P value \| \| <0.0001 \| \| \| P value summary \| \| **** \| \| \| Significant diff. among means (P < 0.05)? \| \| Yes \| \| \| R square \| \| 0.2832 \| \| \| Number of families \| 1 \| \|  \| \|  \|  \|  \|  \| \| Number of comparisons per family \| 6 \| \|  \| \|  \|  \|  \|  \| \| Alpha \| 0.05 \| \|  \| \|  \|  \|  \|  \| \|  \|  \| \|  \| \|  \|  \|  \|  \| \| Tukey's multiple comparisons test \| Mean Diff. \| \| 95.00% CI of diff. \| \| Significant? \| Summary \| Adjusted P Value \|  \| \| Veh vs. Ibr \| 60.60 \| \| 29.89 to 91.32 \| \| Yes \| **** \| <0.0001 \| A-B \| \| Veh vs. PD \| 30.75 \| \| -2.430 to 63.92 \| \| No \| ns \| 0.0791 \| A-C \| \| Veh vs. Cabo \| 40.55 \| \| 9.837 to 71.27 \| \| Yes \| ** \| 0.0048 \| A-D \| \| Ibr vs. PD \| -29.86 \| \| -63.03 to 3.321 \| \| No \| ns \| 0.0928 \| B-C \| \| Ibr vs. Cabo \| -20.05 \| \| -50.76 to 10.67 \| \| No \| ns \| 0.3225 \| B-D \| \| PD vs. Cabo \| 9.806 \| \| -23.37 to 42.98 \| \| No \| ns \| 0.8643 \| C-D \| |
| **Figure 1Q. DYRK1A fluorescence intensity - Cortex** |
| \| \| Table Analyzed \| \| DYRK1A-cortex \| \| --- \| --- \| --- \| \| Data sets analyzed \| \| A-D \| \|  \| \|  \| \| ANOVA summary \| \|  \| \| F \| \| 6.606 \| \| P value \| \| 0.0005 \| \| P value summary \| \| *** \| \| Significant diff. among means (P < 0.05)? \| \| Yes \| \| R square \| \| 0.2090 \| \| Number of families \| 1 \|  \|  \|  \|  \|  \| \| Number of comparisons per family \| 6 \|  \|  \|  \|  \|  \| \| Alpha \| 0.05 \|  \|  \|  \|  \|  \| \|  \|  \|  \|  \|  \|  \|  \| \| Tukey's multiple comparisons test \| Mean Diff. \| 95.00% CI of diff. \| Significant? \| Summary \| Adjusted P Value \|  \| \| Veh vs. Ibr \| 5.440 \| -19.89 to 30.77 \| No \| ns \| 0.9423 \| A-B \| \| Veh vs. PD \| -33.56 \| -58.89 to -8.232 \| Yes \| ** \| 0.0045 \| A-C \| \| Veh vs. Cabo \| -3.098 \| -28.76 to 22.56 \| No \| ns \| 0.9889 \| A-D \| \| Ibr vs. PD \| -39.00 \| -64.33 to -13.67 \| Yes \| *** \| 0.0007 \| B-C \| \| Ibr vs. Cabo \| -8.537 \| -34.20 to 17.12 \| No \| ns \| 0.8181 \| B-D \| \| PD vs. Cabo \| 30.46 \| 4.803 to 56.12 \| Yes \| * \| 0.0134 \| C-D \| \|  \| \| --- \| --- \| --- \| --- \| --- \| --- \| --- \| --- \| --- \| --- \| --- \| --- \| --- \| --- \| --- \| --- \| --- \| --- \| --- \| --- \| --- \| --- \| --- \| --- \| --- \| --- \| --- \| --- \| --- \| --- \| --- \| --- \| --- \| --- \| --- \| --- \| --- \| --- \| --- \| --- \| --- \| --- \| --- \| --- \| --- \| --- \| --- \| --- \| --- \| --- \| --- \| --- \| --- \| --- \| --- \| --- \| --- \| --- \| --- \| --- \| --- \| --- \| --- \| --- \| --- \| --- \| --- \| --- \| --- \| --- \| --- \| --- \| --- \| --- \| --- \| --- \| --- \| --- \| --- \| --- \| --- \| --- \| --- \| --- \| --- \| --- \| --- \| --- \| --- \| --- \| --- \| --- \| --- \| --- \| --- \| --- \| --- \| --- \| --- \| --- \| --- \| --- \| --- \| --- \| --- \| --- \| |
| **Figure 1R. pCDK5 fluorescence intensity - CA1** |
| \| \| Table Analyzed \| \| \| pCDK5- CA1 \| \| \| --- \| --- \| --- \| --- \| --- \| \| Data sets analyzed \| \| \| A-D \| \| \|  \| \| \|  \| \| \| ANOVA summary \| \| \|  \| \| \| F \| \| \| 18.79 \| \| \| P value \| \| \| <0.0001 \| \| \| P value summary \| \| \| **** \| \| \| Significant diff. among means (P < 0.05)? \| \| \| Yes \| \| \| R square \| \| \| 0.4324 \| \| \| Number of families \| 1 \|  \| \|  \| \|  \|  \|  \| \| Number of comparisons per family \| 6 \|  \| \|  \| \|  \|  \|  \| \| Alpha \| 0.05 \|  \| \|  \| \|  \|  \|  \| \|  \|  \|  \| \|  \| \|  \|  \|  \| \| Tukey's multiple comparisons test \| Mean Diff. \| 95.00% CI of diff. \| \| Significant? \| \| Summary \| Adjusted P Value \|  \| \| Veh vs. Ibr \| 22.60 \| 11.86 to 33.34 \| \| Yes \| \| **** \| <0.0001 \| A-B \| \| Veh vs. PD \| 29.05 \| 18.17 to 39.93 \| \| Yes \| \| **** \| <0.0001 \| A-C \| \| Veh vs. Cabo \| 12.85 \| 1.966 to 23.73 \| \| Yes \| \| * \| 0.0141 \| A-D \| \| Ibr vs. PD \| 6.447 \| -4.435 to 17.33 \| \| No \| \| ns \| 0.4093 \| B-C \| \| Ibr vs. Cabo \| -9.755 \| -20.64 to 1.127 \| \| No \| \| ns \| 0.0948 \| B-D \| \| PD vs. Cabo \| -16.20 \| -27.22 to -5.181 \| \| Yes \| \| ** \| 0.0013 \| C-D \| \|  \| \| --- \| --- \| --- \| --- \| --- \| --- \| --- \| --- \| --- \| --- \| --- \| --- \| --- \| --- \| --- \| --- \| --- \| --- \| --- \| --- \| --- \| --- \| --- \| --- \| --- \| --- \| --- \| --- \| --- \| --- \| --- \| --- \| --- \| --- \| --- \| --- \| --- \| --- \| --- \| --- \| --- \| --- \| --- \| --- \| --- \| --- \| --- \| --- \| --- \| --- \| --- \| --- \| --- \| --- \| --- \| --- \| --- \| --- \| --- \| --- \| --- \| --- \| --- \| --- \| --- \| --- \| --- \| --- \| --- \| --- \| --- \| --- \| --- \| --- \| --- \| --- \| --- \| --- \| --- \| --- \| --- \| --- \| --- \| --- \| --- \| --- \| --- \| --- \| --- \| --- \| --- \| --- \| --- \| --- \| --- \| --- \| --- \| --- \| --- \| --- \| --- \| --- \| --- \| --- \| --- \| --- \| --- \| --- \| --- \| --- \| --- \| --- \| --- \| --- \| --- \| --- \| --- \| --- \| --- \| --- \| --- \| --- \| --- \| --- \| --- \| --- \| --- \| --- \| --- \| --- \| --- \| --- \| --- \| --- \| --- \| --- \| --- \| --- \| --- \| --- \| --- \| --- \| --- \| --- \| --- \| --- \| |
| **Figure 1U. Iba-1 fluorescence intensity - Cortex** |
| \| \| Table Analyzed \| \| Iba-1 fluorescence intensity- cortex \| \| \| \| \| --- \| --- \| --- \| --- \| --- \| --- \| \| Data sets analyzed \| \| A-D \| \| \| \| \|  \| \|  \| \| \| \| \| ANOVA summary \| \|  \| \| \| \| \| F \| \| 4.519 \| \| \| \| \| P value \| \| 0.0057 \| \| \| \| \| P value summary \| \| ** \| \| \| \| \| Significant diff. among means (P < 0.05)? \| \| Yes \| \| \| \| \| R square \| \| 0.1514 \| \| \| \| \| Number of families \| 1 \|  \|  \|  \|  \| \|  \| \| Number of comparisons per family \| 6 \|  \|  \|  \|  \| \|  \| \| Alpha \| 0.05 \|  \|  \|  \|  \| \|  \| \|  \|  \|  \|  \|  \|  \| \|  \| \| Tukey's multiple comparisons test \| Mean Diff. \| 95.00% CI of diff. \| Significant? \| Summary \| Adjusted P Value \| \|  \| \| Veh vs. Ibr \| 41.78 \| 11.48 to 72.08 \| Yes \| ** \| 0.0029 \| \| A-B \| \| Veh vs. PD \| 19.74 \| -10.56 to 50.04 \| No \| ns \| 0.3248 \| \| A-C \| \| Veh vs. Cabo \| 26.63 \| -3.667 to 56.93 \| No \| ns \| 0.1050 \| \| A-D \| \| Ibr vs. PD \| -22.04 \| -52.34 to 8.261 \| No \| ns \| 0.2323 \| \| B-C \| \| Ibr vs. Cabo \| -15.15 \| -45.45 to 15.15 \| No \| ns \| 0.5573 \| \| B-D \| \| PD vs. Cabo \| 6.888 \| -23.41 to 37.19 \| No \| ns \| 0.9326 \| \| C-D \| \|  \| \| --- \| --- \| --- \| --- \| --- \| --- \| --- \| --- \| --- \| --- \| --- \| --- \| --- \| --- \| --- \| --- \| --- \| --- \| --- \| --- \| --- \| --- \| --- \| --- \| --- \| --- \| --- \| --- \| --- \| --- \| --- \| --- \| --- \| --- \| --- \| --- \| --- \| --- \| --- \| --- \| --- \| --- \| --- \| --- \| --- \| --- \| --- \| --- \| --- \| --- \| --- \| --- \| --- \| --- \| --- \| --- \| --- \| --- \| --- \| --- \| --- \| --- \| --- \| --- \| --- \| --- \| --- \| --- \| --- \| --- \| --- \| --- \| --- \| --- \| --- \| --- \| --- \| --- \| --- \| --- \| --- \| --- \| --- \| --- \| --- \| --- \| --- \| --- \| --- \| --- \| --- \| --- \| --- \| --- \| --- \| --- \| --- \| --- \| --- \| --- \| --- \| --- \| --- \| --- \| --- \| --- \| --- \| --- \| --- \| --- \| --- \| --- \| --- \| --- \| --- \| --- \| --- \| --- \| --- \| --- \| --- \| --- \| --- \| --- \| --- \| --- \| --- \| --- \| --- \| --- \| --- \| --- \| --- \| --- \| --- \| --- \| --- \| --- \| --- \| --- \| --- \| --- \| --- \| --- \| |
| **Figure 1U. Iba-1-labeled area - Cortex** |
| \| \| Table Analyzed \| Iba-1 labeled area-cortex \| \| --- \| --- \| \|  \|  \| \| Column B \| Ibr \| \| vs. \| vs. \| \| Column A \| Veh \| \|  \|  \| \| Unpaired t test \|  \| \| P value \| 0.0023 \| \| P value summary \| ** \| \| Significantly different (P < 0.05)? \| Yes \| \| One- or two-tailed P value? \| Two-tailed \| \| t, df \| t=3.272, df=38 \| \|  \| \| --- \| --- \| --- \| --- \| --- \| --- \| --- \| --- \| --- \| --- \| --- \| --- \| --- \| --- \| --- \| --- \| --- \| --- \| --- \| --- \| --- \| --- \| --- \| --- \| --- \| --- \| |
| **Figure 1U. Iba-1-positive cell numbers - Cortex** |
| \| \| Table Analyzed \| \| \| Iba-1 posi cells-cortex \| \| \| --- \| --- \| --- \| --- \| --- \| \| Data sets analyzed \| \| \| A-D \| \| \|  \| \| \|  \| \| \| ANOVA summary \| \| \|  \| \| \| F \| \| \| 1.232 \| \| \| P value \| \| \| 0.3040 \| \| \| P value summary \| \| \| ns \| \| \| Significant diff. among means (P < 0.05)? \| \| \| No \| \| \| R square \| \| \| 0.04638 \| \| \| Number of families \| 1 \|  \| \|  \|  \|  \|  \| \| Number of comparisons per family \| 6 \|  \| \|  \|  \|  \|  \| \| Alpha \| 0.05 \|  \| \|  \|  \|  \|  \| \|  \|  \|  \| \|  \|  \|  \|  \| \| Tukey's multiple comparisons test \| Mean Diff. \| 95.00% CI of diff. \| \| Significant? \| Summary \| Adjusted P Value \|  \| \| Veh vs. Ibr \| 73.51 \| -97.77 to 244.8 \| \| No \| ns \| 0.6738 \| A-B \| \| Veh vs. PD \| -44.49 \| -215.8 to 126.8 \| \| No \| ns \| 0.9035 \| A-C \| \| Veh vs. Cabo \| -22.04 \| -193.3 to 149.2 \| \| No \| ns \| 0.9866 \| A-D \| \| Ibr vs. PD \| -118.0 \| -289.3 to 53.27 \| \| No \| ns \| 0.2768 \| B-C \| \| Ibr vs. Cabo \| -95.56 \| -266.8 to 75.73 \| \| No \| ns \| 0.4632 \| B-D \| \| PD vs. Cabo \| 22.45 \| -148.8 to 193.7 \| \| No \| ns \| 0.9859 \| C-D \| \|  \| \| --- \| --- \| --- \| --- \| --- \| --- \| --- \| --- \| --- \| --- \| --- \| --- \| --- \| --- \| --- \| --- \| --- \| --- \| --- \| --- \| --- \| --- \| --- \| --- \| --- \| --- \| --- \| --- \| --- \| --- \| --- \| --- \| --- \| --- \| --- \| --- \| --- \| --- \| --- \| --- \| --- \| --- \| --- \| --- \| --- \| --- \| --- \| --- \| --- \| --- \| --- \| --- \| --- \| --- \| --- \| --- \| --- \| --- \| --- \| --- \| --- \| --- \| --- \| --- \| --- \| --- \| --- \| --- \| --- \| --- \| --- \| --- \| --- \| --- \| --- \| --- \| --- \| --- \| --- \| --- \| --- \| --- \| --- \| --- \| --- \| --- \| --- \| --- \| --- \| --- \| --- \| --- \| --- \| --- \| --- \| --- \| --- \| --- \| --- \| --- \| --- \| --- \| --- \| --- \| --- \| --- \| --- \| --- \| --- \| --- \| --- \| --- \| --- \| --- \| --- \| --- \| --- \| --- \| --- \| --- \| --- \| --- \| --- \| --- \| --- \| --- \| --- \| --- \| --- \| --- \| --- \| --- \| --- \| --- \| --- \| |
| **Figure 1W. Iba-1 fluorescence intensity - CA1** |
| \| \| Table Analyzed \| \| \| Iba-1 fluorescence intensity-CA1 \| \| \| \| \| --- \| --- \| --- \| --- \| --- \| --- \| --- \| \| Data sets analyzed \| \| \| A-D \| \| \| \| \|  \| \| \|  \| \| \| \| \| ANOVA summary \| \| \|  \| \| \| \| \| F \| \| \| 6.773 \| \| \| \| \| P value \| \| \| 0.0004 \| \| \| \| \| P value summary \| \| \| *** \| \| \| \| \| Significant diff. among means (P < 0.05)? \| \| \| Yes \| \| \| \| \| R square \| \| \| 0.2109 \| \| \| \| \| Number of families \| 1 \|  \| \|  \|  \|  \| \|  \| \| Number of comparisons per family \| 6 \|  \| \|  \|  \|  \| \|  \| \| Alpha \| 0.05 \|  \| \|  \|  \|  \| \|  \| \|  \|  \|  \| \|  \|  \|  \| \|  \| \| Tukey's multiple comparisons test \| Mean Diff. \| 95.00% CI of diff. \| \| Significant? \| Summary \| Adjusted P Value \| \|  \| \| Veh vs. Ibr \| 44.37 \| 17.27 to 71.47 \| \| Yes \| *** \| 0.0003 \| \| A-B \| \| Veh vs. PD \| 14.08 \| -13.01 to 41.18 \| \| No \| ns \| 0.5248 \| \| A-C \| \| Veh vs. Cabo \| 27.90 \| 0.8003 to 55.00 \| \| Yes \| * \| 0.0412 \| \| A-D \| \| Ibr vs. PD \| -30.28 \| -57.38 to -3.186 \| \| Yes \| * \| 0.0224 \| \| B-C \| \| Ibr vs. Cabo \| -16.47 \| -43.57 to 10.63 \| \| No \| ns \| 0.3867 \| \| B-D \| \| PD vs. Cabo \| 13.81 \| -13.28 to 40.91 \| \| No \| ns \| 0.5412 \| \| C-D \| \|  \| \| --- \| --- \| --- \| --- \| --- \| --- \| --- \| --- \| --- \| --- \| --- \| --- \| --- \| --- \| --- \| --- \| --- \| --- \| --- \| --- \| --- \| --- \| --- \| --- \| --- \| --- \| --- \| --- \| --- \| --- \| --- \| --- \| --- \| --- \| --- \| --- \| --- \| --- \| --- \| --- \| --- \| --- \| --- \| --- \| --- \| --- \| --- \| --- \| --- \| --- \| --- \| --- \| --- \| --- \| --- \| --- \| --- \| --- \| --- \| --- \| --- \| --- \| --- \| --- \| --- \| --- \| --- \| --- \| --- \| --- \| --- \| --- \| --- \| --- \| --- \| --- \| --- \| --- \| --- \| --- \| --- \| --- \| --- \| --- \| --- \| --- \| --- \| --- \| --- \| --- \| --- \| --- \| --- \| --- \| --- \| --- \| --- \| --- \| --- \| --- \| --- \| --- \| --- \| --- \| --- \| --- \| --- \| --- \| --- \| --- \| --- \| --- \| --- \| --- \| --- \| --- \| --- \| --- \| --- \| --- \| --- \| --- \| --- \| --- \| --- \| --- \| --- \| --- \| --- \| --- \| --- \| --- \| --- \| --- \| --- \| --- \| --- \| --- \| --- \| --- \| --- \| --- \| --- \| --- \| --- \| --- \| --- \| --- \| --- \| --- \| --- \| --- \| --- \| --- \| --- \| --- \| --- \| --- \| --- \| --- \| --- \| --- \| --- \| --- \| |
| **Figure 1W. Iba-1-labeled area - CA1** |
| \| \| Table Analyzed \| \| \| Iba-1 labeled area- CA1 \| \| \| --- \| --- \| --- \| --- \| --- \| \| Data sets analyzed \| \| \| A-D \| \| \|  \| \| \|  \| \| \| ANOVA summary \| \| \|  \| \| \| F \| \| \| 3.669 \| \| \| P value \| \| \| 0.0158 \| \| \| P value summary \| \| \| * \| \| \| Significant diff. among means (P < 0.05)? \| \| \| Yes \| \| \| R square \| \| \| 0.1265 \| \| \| Number of families \| 1 \|  \| \|  \|  \|  \|  \| \| Number of comparisons per family \| 6 \|  \| \|  \|  \|  \|  \| \| Alpha \| 0.05 \|  \| \|  \|  \|  \|  \| \|  \|  \|  \| \|  \|  \|  \|  \| \| Tukey's multiple comparisons test \| Mean Diff. \| 95.00% CI of diff. \| \| Significant? \| Summary \| Adjusted P Value \|  \| \| Veh vs. Ibr \| 2.325 \| 0.2564 to 4.394 \| \| Yes \| * \| 0.0214 \| A-B \| \| Veh vs. PD \| 0.1365 \| -1.932 to 2.205 \| \| No \| ns \| 0.9981 \| A-C \| \| Veh vs. Cabo \| 0.9270 \| -1.142 to 2.996 \| \| No \| ns \| 0.6431 \| A-D \| \| Ibr vs. PD \| -2.189 \| -4.258 to -0.1200 \| \| Yes \| * \| 0.0340 \| B-C \| \| Ibr vs. Cabo \| -1.398 \| -3.467 to 0.6706 \| \| No \| ns \| 0.2931 \| B-D \| \| PD vs. Cabo \| 0.7906 \| -1.278 to 2.859 \| \| No \| ns \| 0.7477 \| C-D \| \|  \| \| --- \| --- \| --- \| --- \| --- \| --- \| --- \| --- \| --- \| --- \| --- \| --- \| --- \| --- \| --- \| --- \| --- \| --- \| --- \| --- \| --- \| --- \| --- \| --- \| --- \| --- \| --- \| --- \| --- \| --- \| --- \| --- \| --- \| --- \| --- \| --- \| --- \| --- \| --- \| --- \| --- \| --- \| --- \| --- \| --- \| --- \| --- \| --- \| --- \| --- \| --- \| --- \| --- \| --- \| --- \| --- \| --- \| --- \| --- \| --- \| --- \| --- \| --- \| --- \| --- \| --- \| --- \| --- \| --- \| --- \| --- \| --- \| --- \| --- \| --- \| --- \| --- \| --- \| --- \| --- \| --- \| --- \| --- \| --- \| --- \| --- \| --- \| --- \| --- \| --- \| --- \| --- \| --- \| --- \| --- \| --- \| --- \| --- \| --- \| --- \| --- \| --- \| --- \| --- \| --- \| --- \| --- \| --- \| --- \| --- \| --- \| --- \| --- \| --- \| --- \| --- \| --- \| --- \| --- \| --- \| --- \| --- \| --- \| --- \| --- \| --- \| --- \| --- \| --- \| --- \| --- \| --- \| --- \| --- \| --- \| |
| **Figure 1W. Iba-1-positive cell numbers - CA1** |
| \| \| Table Analyzed \| Iba-1 posi cells - CA1 \| \| --- \| --- \| \|  \|  \| \| Column B \| Ibr \| \| vs. \| vs. \| \| Column A \| Veh \| \|  \|  \| \| Unpaired t test \|  \| \| P value \| 0.0390 \| \| P value summary \| * \| \| Significantly different (P < 0.05)? \| Yes \| \| One- or two-tailed P value? \| Two-tailed \| \| t, df \| t=2.138, df=38 \| \|  \| \| --- \| --- \| --- \| --- \| --- \| --- \| --- \| --- \| --- \| --- \| --- \| --- \| --- \| --- \| --- \| --- \| --- \| --- \| --- \| --- \| --- \| --- \| --- \| --- \| --- \| --- \| |
| **Figure 1Y. GFAP fluorescence intensity - CA1** |
| \| \| Table Analyzed \| \| \| GFAP- fluorescence intensity- CA1 \| \| \| \| \| --- \| --- \| --- \| --- \| --- \| --- \| --- \| \| Data sets analyzed \| \| \| A-D \| \| \| \| \|  \| \| \|  \| \| \| \| \| ANOVA summary \| \| \|  \| \| \| \| \| F \| \| \| 8.008 \| \| \| \| \| P value \| \| \| 0.0001 \| \| \| \| \| P value summary \| \| \| *** \| \| \| \| \| Significant diff. among means (P < 0.05)? \| \| \| Yes \| \| \| \| \| R square \| \| \| 0.2402 \| \| \| \| \| Number of families \| 1 \|  \| \|  \|  \|  \| \|  \| \| Number of comparisons per family \| 6 \|  \| \|  \|  \|  \| \|  \| \| Alpha \| 0.05 \|  \| \|  \|  \|  \| \|  \| \|  \|  \|  \| \|  \|  \|  \| \|  \| \| Tukey's multiple comparisons test \| Mean Diff. \| 95.00% CI of diff. \| \| Significant? \| Summary \| Adjusted P Value \| \|  \| \| Veh vs. Ibr \| 50.66 \| 22.63 to 78.69 \| \| Yes \| **** \| <0.0001 \| \| A-B \| \| Veh vs. PD \| 14.50 \| -13.52 to 42.53 \| \| No \| ns \| 0.5286 \| \| A-C \| \| Veh vs. Cabo \| 24.71 \| -3.319 to 52.74 \| \| No \| ns \| 0.1034 \| \| A-D \| \| Ibr vs. PD \| -36.15 \| -64.18 to -8.121 \| \| Yes \| ** \| 0.0060 \| \| B-C \| \| Ibr vs. Cabo \| -25.95 \| -53.97 to 2.085 \| \| No \| ns \| 0.0797 \| \| B-D \| \| PD vs. Cabo \| 10.21 \| -17.82 to 38.24 \| \| No \| ns \| 0.7744 \| \| C-D \| \|  \| \| --- \| --- \| --- \| --- \| --- \| --- \| --- \| --- \| --- \| --- \| --- \| --- \| --- \| --- \| --- \| --- \| --- \| --- \| --- \| --- \| --- \| --- \| --- \| --- \| --- \| --- \| --- \| --- \| --- \| --- \| --- \| --- \| --- \| --- \| --- \| --- \| --- \| --- \| --- \| --- \| --- \| --- \| --- \| --- \| --- \| --- \| --- \| --- \| --- \| --- \| --- \| --- \| --- \| --- \| --- \| --- \| --- \| --- \| --- \| --- \| --- \| --- \| --- \| --- \| --- \| --- \| --- \| --- \| --- \| --- \| --- \| --- \| --- \| --- \| --- \| --- \| --- \| --- \| --- \| --- \| --- \| --- \| --- \| --- \| --- \| --- \| --- \| --- \| --- \| --- \| --- \| --- \| --- \| --- \| --- \| --- \| --- \| --- \| --- \| --- \| --- \| --- \| --- \| --- \| --- \| --- \| --- \| --- \| --- \| --- \| --- \| --- \| --- \| --- \| --- \| --- \| --- \| --- \| --- \| --- \| --- \| --- \| --- \| --- \| --- \| --- \| --- \| --- \| --- \| --- \| --- \| --- \| --- \| --- \| --- \| --- \| --- \| --- \| --- \| --- \| --- \| --- \| --- \| --- \| --- \| --- \| --- \| --- \| --- \| --- \| --- \| --- \| --- \| --- \| --- \| --- \| --- \| --- \| --- \| --- \| --- \| --- \| --- \| --- \| |
| **Figure 1Y. GFAP-1-labeled area - CA1** |
| \| \| Table Analyzed \| \| \| GFAP labeled area-CA1 \| \| \| --- \| --- \| --- \| --- \| --- \| \| Data sets analyzed \| \| \| A-D \| \| \|  \| \| \|  \| \| \| ANOVA summary \| \| \|  \| \| \| F \| \| \| 7.144 \| \| \| P value \| \| \| 0.0003 \| \| \| P value summary \| \| \| *** \| \| \| Significant diff. among means (P < 0.05)? \| \| \| Yes \| \| \| R square \| \| \| 0.2200 \| \| \| Number of families \| 1 \|  \| \|  \| \|  \|  \|  \| \| Number of comparisons per family \| 6 \|  \| \|  \| \|  \|  \|  \| \| Alpha \| 0.05 \|  \| \|  \| \|  \|  \|  \| \|  \|  \|  \| \|  \| \|  \|  \|  \| \| Tukey's multiple comparisons test \| Mean Diff. \| 95.00% CI of diff. \| \| Significant? \| \| Summary \| Adjusted P Value \|  \| \| Veh vs. Ibr \| 1.996 \| 0.8091 to 3.183 \| \| Yes \| \| *** \| 0.0002 \| A-B \| \| Veh vs. PD \| 0.5017 \| -0.6852 to 1.689 \| \| No \| \| ns \| 0.6843 \| A-C \| \| Veh vs. Cabo \| 1.038 \| -0.1493 to 2.225 \| \| No \| \| ns \| 0.1079 \| A-D \| \| Ibr vs. PD \| -1.494 \| -2.681 to -0.3074 \| \| Yes \| \| ** \| 0.0077 \| B-C \| \| Ibr vs. Cabo \| -0.9584 \| -2.145 to 0.2285 \| \| No \| \| ns \| 0.1557 \| B-D \| \| PD vs. Cabo \| 0.5359 \| -0.6510 to 1.723 \| \| No \| \| ns \| 0.6375 \| C-D \| \|  \| \| --- \| --- \| --- \| --- \| --- \| --- \| --- \| --- \| --- \| --- \| --- \| --- \| --- \| --- \| --- \| --- \| --- \| --- \| --- \| --- \| --- \| --- \| --- \| --- \| --- \| --- \| --- \| --- \| --- \| --- \| --- \| --- \| --- \| --- \| --- \| --- \| --- \| --- \| --- \| --- \| --- \| --- \| --- \| --- \| --- \| --- \| --- \| --- \| --- \| --- \| --- \| --- \| --- \| --- \| --- \| --- \| --- \| --- \| --- \| --- \| --- \| --- \| --- \| --- \| --- \| --- \| --- \| --- \| --- \| --- \| --- \| --- \| --- \| --- \| --- \| --- \| --- \| --- \| --- \| --- \| --- \| --- \| --- \| --- \| --- \| --- \| --- \| --- \| --- \| --- \| --- \| --- \| --- \| --- \| --- \| --- \| --- \| --- \| --- \| --- \| --- \| --- \| --- \| --- \| --- \| --- \| --- \| --- \| --- \| --- \| --- \| --- \| --- \| --- \| --- \| --- \| --- \| --- \| --- \| --- \| --- \| --- \| --- \| --- \| --- \| --- \| --- \| --- \| --- \| --- \| --- \| --- \| --- \| --- \| --- \| --- \| --- \| --- \| --- \| --- \| --- \| --- \| --- \| --- \| --- \| --- \| |
| **Figure 1Y. GFAP-positive cell numbers - CA1** |
| \| \| Table Analyzed \| \| \| GFAP posi cells - CA1 \| \| \| --- \| --- \| --- \| --- \| --- \| \| Data sets analyzed \| \| \| A-D \| \| \|  \| \| \|  \| \| \| ANOVA summary \| \| \|  \| \| \| F \| \| \| 3.366 \| \| \| P value \| \| \| 0.0229 \| \| \| P value summary \| \| \| * \| \| \| Significant diff. among means (P < 0.05)? \| \| \| Yes \| \| \| R square \| \| \| 0.1173 \| \| \| Number of families \| 1 \|  \| \|  \| \|  \|  \|  \| \| Number of comparisons per family \| 6 \|  \| \|  \| \|  \|  \|  \| \| Alpha \| 0.05 \|  \| \|  \| \|  \|  \|  \| \|  \|  \|  \| \|  \| \|  \|  \|  \| \| Tukey's multiple comparisons test \| Mean Diff. \| 95.00% CI of diff. \| \| Significant? \| \| Summary \| Adjusted P Value \|  \| \| Veh vs. Ibr \| 205.1 \| 10.27 to 400.0 \| \| Yes \| \| * \| 0.0352 \| A-B \| \| Veh vs. PD \| 17.02 \| -177.8 to 211.9 \| \| No \| \| ns \| 0.9957 \| A-C \| \| Veh vs. Cabo \| 24.13 \| -170.7 to 219.0 \| \| No \| \| ns \| 0.9880 \| A-D \| \| Ibr vs. PD \| -188.1 \| -382.9 to 6.749 \| \| No \| \| ns \| 0.0624 \| B-C \| \| Ibr vs. Cabo \| -181.0 \| -375.8 to 13.86 \| \| No \| \| ns \| 0.0782 \| B-D \| \| PD vs. Cabo \| 7.115 \| -187.7 to 202.0 \| \| No \| \| ns \| 0.9997 \| C-D \| \|  \| \| --- \| --- \| --- \| --- \| --- \| --- \| --- \| --- \| --- \| --- \| --- \| --- \| --- \| --- \| --- \| --- \| --- \| --- \| --- \| --- \| --- \| --- \| --- \| --- \| --- \| --- \| --- \| --- \| --- \| --- \| --- \| --- \| --- \| --- \| --- \| --- \| --- \| --- \| --- \| --- \| --- \| --- \| --- \| --- \| --- \| --- \| --- \| --- \| --- \| --- \| --- \| --- \| --- \| --- \| --- \| --- \| --- \| --- \| --- \| --- \| --- \| --- \| --- \| --- \| --- \| --- \| --- \| --- \| --- \| --- \| --- \| --- \| --- \| --- \| --- \| --- \| --- \| --- \| --- \| --- \| --- \| --- \| --- \| --- \| --- \| --- \| --- \| --- \| --- \| --- \| --- \| --- \| --- \| --- \| --- \| --- \| --- \| --- \| --- \| --- \| --- \| --- \| --- \| --- \| --- \| --- \| --- \| --- \| --- \| --- \| --- \| --- \| --- \| --- \| --- \| --- \| --- \| --- \| --- \| --- \| --- \| --- \| --- \| --- \| --- \| --- \| --- \| --- \| --- \| --- \| --- \| --- \| --- \| --- \| --- \| --- \| --- \| --- \| --- \| --- \| --- \| --- \| --- \| --- \| --- \| --- \| |
| **Figure S1. AT180 fluorescence intensity - CA1** |
| \| \| Table Analyzed \| AT180-CA1 \| \| --- \| --- \| \|  \|  \| \| Column B \| Ibr \| \| vs. \| vs. \| \| Column A \| Veh \| \|  \|  \| \| Unpaired t test \|  \| \| P value \| 0.0817 \| \| P value summary \| ns \| \| Significantly different (P < 0.05)? \| No \| \| One- or two-tailed P value? \| Two-tailed \| \| t, df \| t=1.791, df=36 \| \|  \| \| --- \| --- \| --- \| --- \| --- \| --- \| --- \| --- \| --- \| --- \| --- \| --- \| --- \| --- \| --- \| --- \| --- \| --- \| --- \| --- \| --- \| --- \| --- \| --- \| --- \| --- \| |
| **Figure S2. DYRK1A fluorescence intensity - CA1** |
| \| \| Table Analyzed \| \| DYRK1A-CA1 \| \| \| --- \| --- \| --- \| --- \| \| Data sets analyzed \| \| A-D \| \| \|  \| \|  \| \| \| ANOVA summary \| \|  \| \| \| F \| \| 6.105 \| \| \| P value \| \| 0.0009 \| \| \| P value summary \| \| *** \| \| \| Significant diff. among means (P < 0.05)? \| \| Yes \| \| \| R square \| \| 0.1963 \| \| \| Number of families \| 1 \| \|  \|  \|  \|  \|  \| \| Number of comparisons per family \| 6 \| \|  \|  \|  \|  \|  \| \| Alpha \| 0.05 \| \|  \|  \|  \|  \|  \| \|  \|  \| \|  \|  \|  \|  \|  \| \| Tukey's multiple comparisons test \| Mean Diff. \| \| 95.00% CI of diff. \| Significant? \| Summary \| Adjusted P Value \|  \| \| Veh vs. Ibr \| 6.656 \| \| -16.45 to 29.76 \| No \| ns \| 0.8733 \| A-B \| \| Veh vs. PD \| -26.91 \| \| -50.01 to -3.812 \| Yes \| * \| 0.0158 \| A-C \| \| Veh vs. Cabo \| -17.02 \| \| -40.42 to 6.387 \| No \| ns \| 0.2325 \| A-D \| \| Ibr vs. PD \| -33.57 \| \| -56.67 to -10.47 \| Yes \| ** \| 0.0015 \| B-C \| \| Ibr vs. Cabo \| -23.67 \| \| -47.08 to -0.2689 \| Yes \| * \| 0.0464 \| B-D \| \| PD vs. Cabo \| 9.897 \| \| -13.51 to 33.30 \| No \| ns \| 0.6838 \| C-D \| \|  \| \| --- \| --- \| --- \| --- \| --- \| --- \| --- \| --- \| --- \| --- \| --- \| --- \| --- \| --- \| --- \| --- \| --- \| --- \| --- \| --- \| --- \| --- \| --- \| --- \| --- \| --- \| --- \| --- \| --- \| --- \| --- \| --- \| --- \| --- \| --- \| --- \| --- \| --- \| --- \| --- \| --- \| --- \| --- \| --- \| --- \| --- \| --- \| --- \| --- \| --- \| --- \| --- \| --- \| --- \| --- \| --- \| --- \| --- \| --- \| --- \| --- \| --- \| --- \| --- \| --- \| --- \| --- \| --- \| --- \| --- \| --- \| --- \| --- \| --- \| --- \| --- \| --- \| --- \| --- \| --- \| --- \| --- \| --- \| --- \| --- \| --- \| --- \| --- \| --- \| --- \| --- \| --- \| --- \| --- \| --- \| --- \| --- \| --- \| --- \| --- \| --- \| --- \| --- \| --- \| --- \| --- \| --- \| --- \| --- \| --- \| --- \| --- \| --- \| --- \| --- \| --- \| --- \| --- \| --- \| --- \| --- \| --- \| --- \| --- \| --- \| --- \| |

**References**

1. Lee HJ, Jeon SG, Kim J, Kang RJ, Kim SM, Han KM, Park H, Kim KT, Sung YM, Nam HY *et al*: **Ibrutinib modulates Abeta/tau pathology, neuroinflammation, and cognitive function in mouse models of Alzheimer's disease**. *Aging Cell* 2021, **20**(3):e13332.
